# Foot rot tolerant transgenic rough lemon rootstock developed through expression of *β‐1,3‐glucanase* from *Trichoderma* spp.

**DOI:** 10.1111/pbi.13152

**Published:** 2019-06-06

**Authors:** Jagdeep Singh Sandhu, Shivani Nayyar, Ajinder Kaur, Ramanjeet Kaur, Anu Kalia, Anita Arora, Yesmin Kaur, Surinder Kumar Thind, Gautam Chhabra

**Affiliations:** ^1^ School of Agricultural Biotechnology Punjab Agricultural University Ludhiana India; ^2^ Electron Microscopy and Nanoscience Laboratory Punjab Agricultural University Ludhiana India; ^3^ Department of Fruit Science Punjab Agricultural University Ludhiana India; ^4^ Department of Plant Pathology Punjab Agricultural University Ludhiana India

**Keywords:** transgenic rootstock, *Phytophthora parasitica*, hyphal structural degradation, scanning electron microscopy

Citrus (*Citrus* spp.) is grown worldwide commercially for consumption as fresh fruit and squeezed juice. The crop is most vulnerable to fungal pathogen *Phytophthora parasitica* that attacks citrus trees of all ages and incites foot rot disease, also known as gummosis causing significant fruit yield losses (Naqvi, 2004). The infection occurs by entry of motile zoospores from soil into root cortex, thus decaying fibrous roots, and upon splashing of zoospores on trunk bark near the ground leading to entry in bark cambium through wounds or cracks resulting in exudation of gum from cracks, girdling of bark cambium and eventually killing the tree (Graham and Timmer, 1994). *Phytophthora* infection can be managed by soil drainage, alleviated through application of systemic fungicides, though not always cost‐effective and environment‐friendly (Naqvi, 2004). The pathogen infection has also been controlled by the use of biological agents, such as *Trichoderma* spp. that acts by producing various cell wall‐degrading enzymes; among these, β‐1,3‐glucanase hydrolyses β‐1,3‐glucosyl linkages of β‐glucan‐rich (90%) *Phytophthora* cell wall, resulting in inhibition of pathogen hyphae and restriction of disease development (Bartnicki‐Garcia, 1966; Benítez *et al*., 2004). Cloning of β‐1,3‐glucanase encoding genes, such as *bgn*13.1, *gluc*78, *Tv‐bgn1* and *Tv‐bgn2* from *Trichoderma* spp., and expression in transgenic plants confirmed their role in providing resistance against fungal pathogens (Benítez *et al*., 2004). There is no information in literature about the expression of *β‐1*,*3‐glucanase* from *Trichoderma* in citrus for imparting resistance against *Phytophthora* spp.

Citrus crop is grown as composite trees with scion grafted onto a rootstock; the main reason for shifting citriculture from seedling to grafted plants was major losses caused by foot rot (Castle, 2010). Rootstocks exert strong influence on treesꞌ ability to confront stress, besides determining fruit yield and quality; however, every rootstock has certain unfavourable traits that preclude its universal use. Rough lemon (*C. jambhiri*) is a widely used rootstock possessing most desirable traits with ability to form congenial scionic combinations, but is highly susceptible to foot rot (Naqvi, 2004; Castle, 2010).

This study was undertaken to develop foot rot tolerant transgenic rough lemon rootstock through the expression of *β‐1,3‐glucanase* isolated from *Trichoderma* spp. The gene cassette carrying *β‐1,3‐glucanase* catalytic domain driven by CaMV 35S promoter and NOS terminator in pBI121 was mobilized into *Agrobacterium* strain LBA 4404 (Figure 1a). The transgene was expressed constitutively as *Phytophthora* is persistently present in soil, infecting roots and trunk at various stages of plant development (Naqvi, 2004). *Agrobacterium* transformed epicotyl segments exhibited shoot emergence in clusters on cut edges within 8 weeks, and 188 T_0_ plants were transferred to soil (Figure 1b–f). The presence of catalytic domain was verified in 13 putative plants through PCR; the absence of *virG* amplicon substantiated that these plants were not false positives contaminated by *Agrobacterium*. The transcription of transgene in 13 putative plants through semi‐quantitative RT‐PCR demonstrated successful uptake of T‐DNA by host plant cells (Figure 1g). The relative transgene expression in RT‐PCR positive plants analysed through qRT‐PCR revealed 2.81, 2.51, 2.58, 2.67, 3.25, 1.87 and 3.55‐fold higher expression in seven transgenic plants designated as RL‐5, RL‐7, RL‐12, RL‐16, RL‐17, RL‐60 and RL‐78, respectively, as compared to NT plant (Figure 1p), whereas expression in six plants RL‐79, RL‐96, RL‐117, RL‐120, RL‐149 and RL‐165 was comparable to NT plant. The total proteins extracted from 20‐week‐old transgenic and NT plants were confronted *in vitro* with *P. parasitica* to test their antifungal activity. The results revealed inhibition of *Phytophthora* mycelial growth (20.49–31.69%) by transgenic plant proteins, as compared to no restrain on pathogen by NT proteins (Figure 1h). The scanning electron micrographs of mycelia inhibited by transgenic proteins displayed hyphal swelling (Figure 1i), cytoplasmic constrictions, termination of mycelial branches (Figure 1j) followed by fragmentation (Figure 1k) confirming structural degradation of mycelium, whereas mycelia around NT plant proteins were normal displaying unswollen hyphae (Figure 1l). The results pointed that recombinant protein might be acting as an impediment against *Phytophthora*. The antifungal action of β‐1,3‐glucanase enzymes BGN13.1 and GLUC78 from *Trichoderma* spp. is documented against a range of fungi including *Phytophthora* (de la Cruz *et al*., 1995).

**Figure 1:**
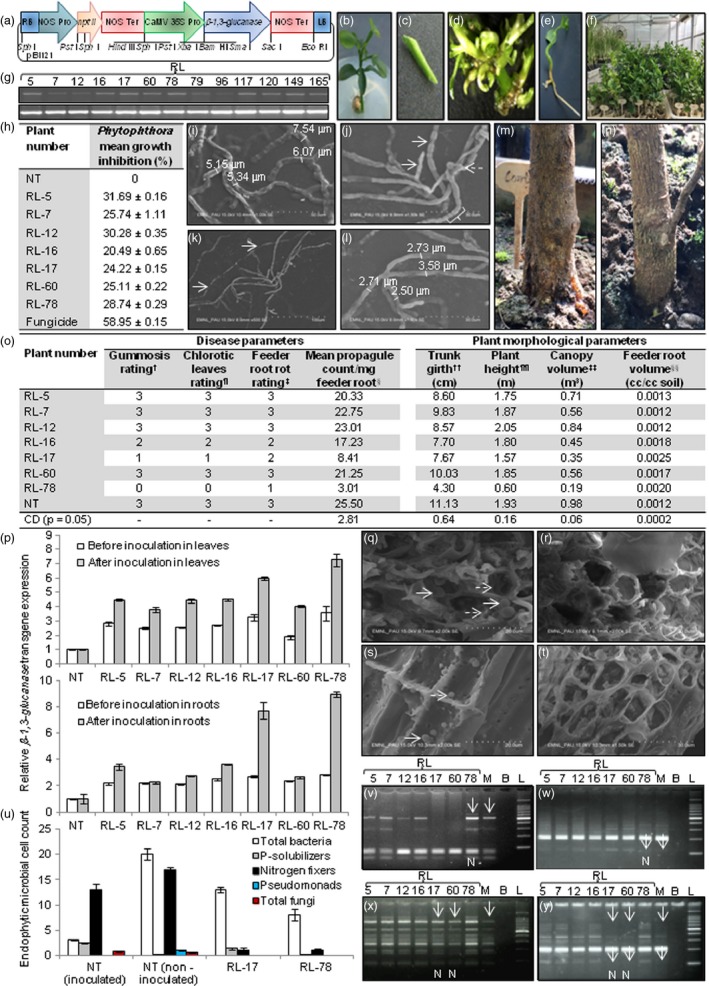
Foot rot tolerant transgenic rough lemon rootstock. (a) Map of gene cassette carrying 1764‐bp β*‐1,3‐glucanase* catalytic domain (KJ603460). (b) *In vitro‐*raised seedling. (c) Epicotyl segment. (d) Direct regeneration. (e) Root formation. (f) T_0_ plants. (g) Semi‐quantitative RT‐PCR using catalytic domain primers 5ꞌ‐ATGTTGAAGCTCACGGCGCTCGTTG‐3ꞌ and 5ꞌ‐ACTCGATTGCAGGGAAAGGCGGA‐3ꞌ (top panel), RL‐5 to RL‐165 denote transgenic plants; cDNA integrity using *26SrRNA* primers 5ꞌ‐CACAATGATAGGAGGAGCCGAC‐3ꞌ and 5ꞌ‐CAAGGGAACGGGCTTGGCAGAATC‐3ꞌ (AY283368) [bottom panel]. (h) *Phytophthora* mycelial growth inhibition by transgenic plant proteins; growth inhibition (%) = [(growth in NT ˗ growth in RL)/growth in NT] × 100; the assay was performed in triplicate; results are presented as mean ± SE. (i) Swollen hyphae; numbers indicate hyphal thickness. (j) Hyphal cytoplasmic constrictions with beaded appearance (solid arrows, bracket) and termination of mycelial branches (broken arrow). (k) Hyphal fragmentation; arrows indicate break points. (l) Normal unswollen hyphae. (m) *Phytophthora*‐inoculated NT plant displaying gumming symptoms. (n) Inoculated transgenic plant RL‐78 with the absence of gumming. (o) Estimation of foot rot incidence and plant morphological parameters: ^†^Gumming scale 0–4, where 0 = nil, 1 = slight gumming, 2 = moderate gumming, 3 = severe gumming, 4 = tree girdled; ^¶^leaf chlorosis scale 0–3, where 0 = no symptoms, 1 = chlorotic leaves (light symptoms), 2 = necrotic leaves (moderate symptoms), 3 = wilt and defoliation (severe symptoms); ^‡^feeder root rot scale 1–5, where 1 = no visible symptoms, 2 = a few roots (1–25%) with rotting symptoms, 3 = majority of roots (26–50%) with rotting symptoms, 4 = all roots infected (51–75% rotting), major roots dead, 5 = majority of roots (>76% rotting) dead or missing; ^§^propagule count was mean of three replicates; ^††^trunk girth was assessed at 20 cm above ground; ^¶¶^plant height was measured from ground to highest point; ^‡‡^canopy volume = 0.5236 × plant height × canopy spread (north to south) × canopy spread (east to west); ^§§^feeder root volume = mean feeder root volume/total soil volume, where total soil volume = 141.14 cc [Π × (radius of cylinder 1.59 cm)^2^ × height of cylinder 17.78 cm]; plant morphological parameters were recorded thrice, and *P *<* *0.05 was statistically significant. (p) Quantitative RT‐PCR in leaves, roots of transgenic plants before and after *Phytophthora* inoculation using qRT‐PCR *β‐1,3‐glucanase* primers 5ꞌ‐CTCTCCAGAATGCTATCACCAC‐3ꞌ and 5ꞌ‐GGTATATGTTCCCGGAGGAATG‐3ꞌ; *18SrRNA* reference gene primers 5ꞌ‐TCGGGTGTTTTCACGTCTCA‐3ꞌ and 5ꞌ‐TGGATGCCGCTGGGAAGC‐3ꞌ (FJ356261.1); error bars represent range of change in *β‐1,3‐glucanase* expression determined by 2^−∆∆C^_T_ method. (q) Transverse root section of susceptible NT plant revealing the occurrence of ligni‐tubers (solid arrows) and callose deposition (broken arrows) in cortex cells. (r) The absence of ligni‐tubers and callose deposition in cortex cells of tolerant plant RL‐78. (s) The occurrence of zoospores on side wall (solid arrow) and end plate (broken arrow) of root xylem cell from susceptible NT plant. (t) The absence of zoospores in xylem cells of tolerant plant RL‐78. (u) Endophytic microbial cell count in roots of transgenic rough lemon plants inoculated with *Phytophthora*; the microbial cell count (10^3^ cfu/mL) was recorded in triplicate, and results are presented as mean ± SE. (v) Allelic pattern using CCSM‐17 (5ꞌ‐ACATGGACAGGACAACTAAG‐3ꞌ and 5ꞌ‐GTTATGATACGTCTGTGTTCC‐3ꞌ); arrows indicate the presence of additional allele, RL‐5 to RL‐78 denotes transgenic plants, M refers to mother plant, B represents blank lane, L denotes 100‐bp DNA ladder (Promega, Cat. No. G2101), and N indicates nucellar plant. (w) CCSM‐6 (5ꞌ‐ATCTGTGTGAGGACTGAA‐3ꞌ and 5ꞌ‐CCTCTATTAATGTGCCTG‐3ꞌ); arrows depict the absence of allele. (x) CCSM‐13 (5ꞌ‐CTAGAGCCGAATTCACC‐3ꞌ and 5ꞌ‐AACAGCTACCAAGACACC‐3ꞌ); arrows show the absence of allele. (y) CCSM‐18 (5ꞌ‐GTGATTGCTGGTGTCGTT‐3ꞌ and 5ꞌ‐AACAGTTGATGAAGAGGAAG‐3ꞌ); arrows specify the absence of alleles.

The 18‐month‐old transgenic and NT plants were bioassayed for foot rot incidence by injecting *P. parasitica* inoculum in trunk (30 μL, 4 × 10^4^ zoospores/mL) and inoculation of plant rhizospheric soil (20 propagules/cc), as the pathogen propagules >10/cc soil are considered destructive (Naqvi, 2004). NT plant displayed susceptibility symptoms after 12 weeks of inoculation revealing severe gumming on trunk (Figure 1m), wilting and leaf defoliation, low feeder root volume due to 50% root rot with high pathogen propagule count/mg root, whereas transgenic plant RL‐78 did not exhibit gumming (Figure 1n) with leaf chlorosis with had high feeder root volume with no visible root rot and significantly low pathogen propagule count (Figure 1o), pointing towards tolerance to *Phytophthora*. RL‐17 showed slight gumming on trunk, light leaf chlorosis, high feeder root volume with a few roots (<25%) showing rotting and low pathogen propagule count, indicating moderate tolerance of plant towards the pathogen. RL‐16 demonstrated moderate gumming on trunk, leaf necrosis, low feeder root volume with up to 25% root rot and high pathogen propagule count, evincing its moderate susceptibility to *Phytophthora*. The plants RL‐5, RL‐7, RL‐12 and RL‐60 were susceptible. Resistance against fungal pathogens through expression of *β‐1,3‐glucanase* from *Trichoderma* spp. has been demonstrated in transgenic rice, pearl millet, canola, strawberry and sugarcane (Nayyar *et al*., 2017).

The comparison of relative *β‐1,3‐glucanase* expression in transgenic plants through qRT‐PCR before inoculation and 12 weeks after pathogen inoculation revealed that transgene expression increased twofold in leaves and threefold in roots of RL‐17 and RL‐78 after inoculation (Figure 1p). The level of up‐regulation was more in tolerant plants as compared to susceptible and NT plants, suggesting involvement of transgene in imparting tolerance against foot rot. *β‐1,3‐glucanase* induction upon pathogen infection and its role in defence were substantiated in transgenic tobacco (Castresana *et al*., 1990).

Scanning electron microscopy of root cortex, xylem cells from *Phytophthora*‐inoculated tolerant transgenic RL‐78 and susceptible NT plants was carried out to observe pathogen invasion. The cortex cells of susceptible NT plant revealed the occurrence of digitate ligni‐tubers and callose deposition (Figure 1q), whereas these entities were absent in tolerant plant RL‐78 in response to *Phytophthora* inoculation (Figure 1r). The xylem cells of susceptible NT plant demonstrated the occurrence of zoospores (Figure 1s); on the contrary, zoospores were absent in xylem tissue of RL‐78 (Figure 1t). The results confirmed that *Phytophthora* could not invade root cells of tolerant transgenic plant RL‐78 due to sloughing off root cells; sloughed off cells usually discharge β‐1,3‐glucanase among other antimicrobial proteins in rhizosphere that remain effective and stable even in the presence of active microflora with ability to bind tightly on plant root surface (Glandorf *et al*., 1997). The lignification of root cells in NT plant could not provide protection against *Phytophthora*, implying non‐effectiveness of native plant defence mechanism. The non‐effectiveness of lignin deposition in restricting pathogen growth was also shown in avocado rootstock root cells upon *Phytophthora* infection (van den Berg *et al*., 2018).

The root tissue of tolerant RL‐78, moderately tolerant RL‐17, susceptible NT plants after *Phytophthora* inoculation and noninoculated NT plant was evaluated for viable endophytic microbial cell count. The susceptible NT plant had highest count of P solubilizers, total fungi and substantial nitrogen fixers (Figure 1u). The non‐inoculated NT plant exhibited highest count of total bacteria, nitrogen fixers, pseudomonads and considerable total fungi cell populations. RL‐78 and RL‐17 did not show the presence of pseudomonads, total fungi and had meagre count of P solubilizers with nitrogen fixers. The results indicated reduction in count of endophytic bacteria and total fungi inhabiting roots of tolerant plants. The constitutive *β‐1,3‐glucanase* expression in transgenic plants has been shown to reduce mycorrhizal symbiosis and rhizospheric microbial diversity due to alteration in structures of symbiotic microbes, and these have a role in plant growth stimulation (Glandorf *et al*., 1997). *Phytophthora* tolerant RL‐78 exhibited slow growth as compared to moderately tolerant RL‐17, moderately susceptible RL‐16 and NT plants (Figure 1o), demonstrating inverse correlation between level of tolerance and plant development rate. The genotyping of transgenic plants revealed that allelic pattern of RL‐78 generated using SSR marker CCSM‐17 was homologous with the mother plant and homology was reaffirmed by CCSM‐6 (Figure 1v,w). The allelic pattern in RL‐17 and RL‐60 using CCSM‐13 revealed their similarity to each other and mother plant; the same was confirmed by CCSM‐18 (Figure 1x,y). The transgenic plants sharing genetic resemblance with the mother plant were identified as nucellar.

*Phytophthora* tolerance in transgenic rough lemon was due to fragmentation of pathogen hyphae on rhizoplane that occurred through direct action of β‐1,3‐glucanase protein on β‐1,3‐glucosyl linkages of *Phytophthora* cell wall. In addition, indirect action of β‐1,3‐glucanase is hypothesized through liberation of oligosaccharide elicitors from fungal cell walls that elicit plant defence response. Thus, it is anticipated that a combination of protective activities initiated upon pathogen infection, and further studies may disclose the composite *β‐1,3‐glucanase*‐mediated tolerance mechanism.

## References

[pbi13152-bib-0001] Bartnicki‐Garcia, S. (1966) Chemistry of hyphal walls of *Phytophthora* . J. Gen. Microbiol. 42, 57–69. 5922299 10.1099/00221287-42-1-57

[pbi13152-bib-0002] Benítez, T. , Rincón, A.M. , Limón, M.C. and Codón, A.C. (2004) Biocontrol mechanisms of *Trichoderma* strains. Int. Microbiol. 7, 249–260. 15666245

[pbi13152-bib-0003] van den Berg, N. , Christie, J.B. , Aveling, T.A.S. and Engelbrecht, J. (2018) Callose and β‐1,3‐glucanase inhibit *Phytophthora cinnamomi* in a resistant avocado rootstock. Plant. Pathol. 67, 1150–1160.

[pbi13152-bib-0004] Castle, W.S. (2010) A career perspective on citrus rootstocks, their development and commercialization. HortScience, 45, 11–15.

[pbi13152-bib-0005] Castresana, C. , Carvalho, F.D. , Gheysen, G. , Habets, M. , Inze, D. and Montagu, M.V. (1990) Tissue‐specific and pathogen‐induced regulation of a *Nicotiana plumbaginifolia* β‐1,3‐glucanase gene. Plant Cell, 2, 1131–1143. 2152158 10.1105/tpc.2.12.1131 PMC159961

[pbi13152-bib-0006] de la Cruz, J. , Pintor‐Toro, J.A. , Benítez, T. , Liobell, A. and Romero, L.C. (1995) A novel endo‐beta‐1,3 glucanase, BGN13.1, involved in the mycoparasitism of *Trichoderma harzianum* . J. Bacteriol. 177, 6937–6945. 7592488 10.1128/jb.177.23.6937-6945.1995 PMC177563

[pbi13152-bib-0007] Glandorf, D.C.M. , Bakker, P.A.H.M. and Van Loon, L.C. (1997) Influence of the production of antibacterial and antifungal proteins by transgenic plants on the saprophytic soil microflora. Acta Bot. Neerl. 46, 85–105.

[pbi13152-bib-0008] Graham, J.H. and Timmer, L.W. (1994) Phytophthora diseases of citrus. http://edis.ifas.ufl.edu.

[pbi13152-bib-0009] Naqvi, S.A.M.H. (2004) Diagnosis and management of certain important fungal diseases of citrus In Diseases of Fruits and Vegetables (Naqvi S.A.M.H., ed), pp. 247–290. Netherlands: Kluwer Academic Publishers.

[pbi13152-bib-0010] Nayyar, S. , Sharma, B.K. , Kaur, A. , Kalia, A. , Sanghera, G.S. , Thind, K.S. , Yadav, I.S. *et al* (2017) Red rot resistant transgenic sugarcane developed through expression of *β‐1*,*3‐glucanase* gene. PLoS ONE, 12, e0179723. 28658312 10.1371/journal.pone.0179723 PMC5489175

